# Structural and functional characterization of Cas2 of CRISPR-Cas subtype I-C lacking the CRISPR component

**DOI:** 10.3389/fmolb.2022.988569

**Published:** 2022-09-12

**Authors:** Vineet Anand, Harshini Sheeja Prabhakaran, Prerana Gogoi, Shankar Prasad Kanaujia, Manish Kumar

**Affiliations:** Department of Biosciences and Bioengineering, Indian Institute of Technology Guwahati, Guwahati, Assam, India

**Keywords:** Leptospira, CRISPR-Cas, endodeoxyribonuclease, ribonuclease, deoxyribonuclease

## Abstract

The genome of pathogenic *Leptospira interrogans* serovars (Copenhageni and Lai) are predicted to have CRISPR-Cas of subtypes I-B and I-C. Cas2, one of the core Cas proteins, has a crucial role in adaptive defense against foreign nucleic acids. However, subtype I-C lacks the CRISPR element at its loci essential for RNA-mediated adaptive immunity against foreign nucleic acids. The reason for sustaining the expense of cas genes are unknown in the absence of a CRISPR array. Thus, Cas2C was chosen as a representative Cas protein from two well-studied serovars of *Leptospira* to address whether it is functional. In this study, the recombinant Cas2C of *Leptospira* serovars Copenhageni (rLinCas2C, 12 kDa) and Lai (rLinCas2C_Lai, 8.6 kDa) were overexpressed and purified. Due to natural frameshift mutation in the cas2c gene of serovar Lai, rLinCas2C_Lai was overexpressed and purified as a partially translated protein. Nevertheless, the recombinant Cas2C from each serovar exhibited metal-dependent DNase and metal-independent RNase activities. The crystal structure of rLinCas2C obtained at the resolution of 2.60 Å revealed the protein is in apostate conformation and contains N- (1–71 amino acids) and C-terminal (72–90 amino acids) regions, with the former possessing a ferredoxin fold. Substitution of the conserved residues (Tyr7, Asp8, Arg33, and Phe39) with alanine and deletion of Loop L2 resulted in compromised DNase activity. On the other hand, a moderate reduction in RNase activity was evident only in selective rLinCas2C mutants. Overall, in the absence of an array, the observed catalytic activity of Cas2C may be required for biological processes distinct from the CRISPR-Cas-associated function.

## Introduction

The genus *Leptospira* is a spiral-shaped bacteria, the pathogenic species of which are known for causing leptospirosis disease in humans and a wide range of animals (Faine, 1974). In nature, the genus *Leptospira* exists in pathogenic, intermediate, and saprophytic forms. These forms can be classified into 26 serogroups and over 300 serovars (Guglielmini et al., 2019). The genome of two well-studied pathogenic leptospires (*L. interrogans* serovars Copenhageni and Lai) harbors genetic elements of an adaptive defense system against foreign nucleic acids known as clustered regularly interspaced short palindromic repeats and their associated genes (CRISPR-Cas) (Fouts et al., 2016).

CRISPR-Cas systems involved in combatting exotic nucleic acids are cataloged into two classes, six types, and thirty-three subtypes according to the association of the signature *cas* genes (Makarova et al., 2019). Based on the signature *cas* genes, the CRISPR-Cas type I system has been classified into seven subtypes (I-A, I-B, I-C, I-D, I-E, I-F, and I-U) (Makarova et al., 2019). The CRISPR-Cas is composed of a CRISPR array preceded by an AT-rich leader sequence and a set of effector *cas* genes encoding nucleases (Jansen et al., 2002). In the genome of pathogenic leptospires (*L. interrogans* serovars Copenhageni and Lai), there are two predetermined subtypes (I-B and I-C) of CRISPR-Cas (Makarova et al., 2015). In *Leptospira*, the CRISPR-Cas subtype I-C lacks the CRISPR array component and is thus considered an orphan CRISPR-Cas system (Xiao et al., 2019). Therefore, it was interesting to investigate whether *C*RISPR-*as*sociated genes (*cas*) of subtype I-C (*cas1* to *cas8*) of *Leptospira,* which lack an array component, are functionally active. Thus, in our preliminary study, out of the eight *cas* genes, we chose *cas2* to clone, overexpress and check the nuclease activity of the purified recombinant protein.

The Cas2 proteins (80–120 residues) are core metallonucleases found universally in all CRISPR-bearing taxa (Samai et al., 2010). Although the Cas2 proteins are not involved in synthesizing the pre-crRNAs or their processing, the genetic studies signify their role in framing the initial stage (adaptation) of immunity against exotic nucleic acids (Yosef et al., 2012; Nuñez et al., 2014). The structural and functional characterization of several Cas2 orthologs has been conducted; however, the catalytic role of Cas2 in CRISPR biology is not well-illustrated to date (Beloglazova et al., 2008; Samai et al., 2010; Kwon et al., 2012; Nam et al., 2012; Ka et al., 2014; Jung et al., 2016). The tertiary structure of pure Cas2 from various organisms, including SsoCas2 (*Sulfolobus solfataricus*), BhaCas2 (*Bacillus halodurans*), SpyCas2 (*Streptococcus pyogenes*), DvuCas2 (*Desulfovibrio vulgaris*), and TonCas2 (*Thermococcus onnurineus*) contains N- and C-terminal regions, with the former having a ferredoxin (βαββαβ) fold (Beloglazova et al., 2008; Samai et al., 2010; Nam et al., 2012; Ka et al., 2014; Jung et al., 2016). The pure SsoCas2, SpyCas2, BhaCas2, DvuCas2, and TonCas2 form a dimer by the interaction of the β5 strand of each subunit at the C-termini (Beloglazova et al., 2008; Samai et al., 2010; Nam et al., 2012; Ka et al., 2014; Jung et al., 2016). In the SsoCas2 dimer, a pair of conserved aspartate residues (Asp10) are involved in catalytic activity (Beloglazova et al., 2008). In *E. coli,* one dimeric unit of Cas2 interacts with the two units of Cas1 dimers to form a heterohexameric complex. Henceforth, Cas2 of *E. coli* facilitates the acquisition of exotic nucleic acid (protospacers) non-catalytically into the CRISPR array (Nuñez et al., 2014; Rollie et al., 2015; Lee et al., 2019). The active-site mutation of Cas2 does not abolish the spacer acquisition (adaptation) by the heterohexameric complex of Cas1-Cas2 in *E. coli.* Thus, the biological significance of Cas2 catalytic activity is equivocal in CRISPR biology. Indeed, the Cas2 of *E. coli* acts non-catalytically as a yardstick to gauge the protospacer length, while Cas1 functions as an integrase (endonuclease) on the cut and paste mechanism (Wang et al., 2015). However, the catalytic activity of Cas2 has been associated with the virulence process in *Legionella pneumophila*, the causative agent of Legionnaires’ disease (Gunderson et al., 2015). Among other functions, Cas2 is also associated with morphological changes in *E. coli* (Wang et al., 2019). Thus, the catalytic activity of Cas2 in bacteria may be utilized for biological processes distinct from the CRISPR-Cas-associated function.

In the genome of pathogenic *L. interrogans* serovar Copenhageni (LinCas2B, ORF id: LIC10941 and LinCas2C, ORF id: LIC12917) and *L. interrogans* serovar Lai (LinCas2B_Lai, ORF id: LA3182 and LinCas2C_Lai, ORF id: LA0683), there are two Cas2 proteins, each in the locus of CRISPR-Cas subtypes I-B and I-C, respectively. Although LinCas2B (LIC10941) and LinCas2B_Lai (LA3182) shared an identical protein sequence, the sequence similarity between LinCas2C (LIC12917) and LinCas2B (LIC10941) is 32%. Moreover, *cas2c* (*LA0683*) in *Leptospira* serovar Lai encodes only the first 58 amino acids (LinCas2C_Lai) because of the natural frameshift mutation (Xiao et al., 2019). It was thus interesting to decipher the nuclease activity in the naturally truncated LinCas2C_Lai (LA0683) protein.

In this study, we sought to characterize the recombinant Cas2C protein of serovars Copenhageni and Lai and compared its activity with well-characterized LinCas2B. Unlike Cas2 from other organisms, the purified rLinCas2C and rLinCas2C_Lai exhibited metal-dependent DNase and metal-independent RNase activity. The determined crystal structure of rLinCas2C ascertained its existence in the dimeric form with the characteristic N-terminal ferredoxin fold (βαββαβ) and was further compared with its homologs. This is the first report concerning the crystal structure of CRISPR-Cas elements from spirochetes.

## Materials and methods

### Bioinformatics analysis

Nucleotide sequences of CRISPR-Cas I-C harbored in *L. interrogans* serovars Copenhageni and Lai were retrieved from NCBI. The three-dimensional (3D) atomic coordinates of the Cas2 orthologs were downloaded from the Protein Data Bank (PDB) (Berman et al., 2002). The genetic architecture of CRISPR-Cas I-C was created based on the *cas* gene coordinates previously documented (Makarova et al., 2015) and using the CRISPRone program (Zhang and Ye, 2017). The phylogenetic tree was constructed by the maximum likelihood method and bootstrapped (1,000 replicates) to evaluate the reliability of the tree generated using the program MEGA11 (Tamura et al., 2013). The 3D structures of LinCas2C_Lai and LinCas2B were predicted using the programs I-TASSER (Yang and Zhang, 2015), Phyre2 (Kelley et al., 2015), and the Swiss model (Biasini et al., 2014). The predicted model’s energy was minimized and then refined using the web server ModRefiner (Xu and Zhang, 2011). Multiple sequence alignment was conducted using the program Clustal Omega (Sievers et al., 2011) with the default set of parameters and decorated using the web tool ESPript for better visual effect (Gouet et al., 2003). Molecular docking of LinCas2C with a non-specific dsDNA was performed using the program NPDock (Tuszynska et al., 2015). The program PyMOL (DeLano, 2002) was used to generate the superimposition of structures. The polar contacts between LinCas2C protomers and the LinCas2C-DNA interface were identified within a distance radius of 3.5 Å. The buried surface area of the Cas2C dimer was calculated using the webserver PDBePISA (Velankar et al., 2010). LinCas2C with Mg^2+^ ion was modeled using *Enterococcus faecalis* Cas1-Cas2/prespacer ternary complex as a template (PDB id: 5XVP) (Xiao et al., 2017).

### Nucleic acid isolation and cloning

The spirochete *L. interrogans* serovar Copenhageni strain Fiocruz L1-130 or serovar Lai culture was maintained in Ellinghausen-McCullough-Johnson-Harris (EMJH) media at 29°C supplemented with 1×enrichment media (Difco) along with 5-fluorouracil (100 μg/ml). After 7 days of incubation, the grown culture was sub-cultured successively. Genomic DNA of *L. interrogans* serovars Copenhageni and Lai were isolated from a 7-day-old culture containing ∼10^8^ cells per ml using QIAamp DNA Blood Mini Kit (Qiagen) per manufacturer protocol. *E. coli* strains DH5α and BL21 (DE3) were grown in Luria Bertani (LB, Himedia) broth or agar for cloning, transformation, and expression.

The open reading frame (ORF) of *LIC12917* (*cas2c*, 273 bp) and *LA0683* (*cas2c_Lai,* 272 bp) were amplified using the genomic DNA templates of *L. interrogans* serovars Copenhageni and Lai, respectively. Both full-length *cas2c* genes were cloned in the pCDF-1b expression vector (Novagen), and cloning was confirmed by double digestion of insert (*BamH*I-*Sal*I) and sequencing of plasmids.

### Nuclease activity assay

Nuclease activity of rLinCas2C was investigated on various DNA and RNA substrates. RNA transcript of the *luciferase* gene was synthesized using HiScribe T7 high yield RNA synthesis kit (NEB) as per the manufacturer protocol. The plasmid was isolated from a 5 ml overnight grown culture of *E. coli* DH5α cells using a mini-prep kit (Thermo Scientific). Single-stranded viral DNA substrate (M13mp18, Фx174) and all enzymes used for genetic engineering were purchased commercially (NEB or Fermentas). As previously reported, short DNA oligomers of 23-mer and 50-mer were used (Rollie et al., 2015). The substrates used for nuclease activity of rLinCas2C were circular double-stranded (ds) plasmid DNA (pET28a, pTZ57R/T, 0.5 µg), circular single-stranded (ss) DNA (M13mp18, 0.5 µg), linear ssDNA (Φx174 genome, 0.5 µg), 23- and 50-mer nucleotides (0.4 µM), and firefly *luciferase* mRNA (0.5 µg) (Supplementary Table S1). The given amount of each substrate was independently incubated with rLinCas2C (25 μM) in a total reaction volume of 25 μL of nuclease buffer (25 mM Tris-HCl pH 8.0, 100 mM KCl, and 2.5 mM MgCl_2_) for an hour at 37°C. DNase activity dependence for divalent metal ions (2.5 mM) was determined by substituting various divalent metal ions (MgCl_2_, MnSO_4_, CaCl_2_, NiSO_4_, FeSO_4_, CuSO_4_, and ZnSO_4_). All the reaction products were separated on ethidium bromide-stained 2% (w/v) agarose gel electrophoresis. The nuclease reaction containing 23- and 50-mer nucleotides were assessed on 8 M 15% urea-PAGE.

### Site-directed mutagenesis

Using the Q5 site-directed mutagenesis kit (NEB), rLinCas2C mutant variants were generated. The mutants were generated using the template plasmid pCDF_LIC12917 and the primers used are listed in Supplementary Table S1. In rLinCas2C, potential residues involved in nuclease activities were substituted with alanine at one or multiple sites to generate various mutant variants (rLinCas2C^Y7A^, rLinCas2C^Y7A+D8A^, rLinCas2C^R33A+F39A^, and rLinCas2C^Y7A+D8A+R33A+F39A^). In one of the mutant variants (rLinCas2C^ΔL2^), residues involved in framing the loop L2 were deleted. All the generated constructs were outsourced for sequencing before overexpression, purification, and characterization of proteins.

Quantitative RNase activity of rLinCas2C, rLinCas2C_Lai, and the mutant variants of rLinCas2C was done using the RNaseAlert kit (Integrated DNA technology, IDT; Cat # 11-02-01-02). The RNaseAlert kit contains synthetic RNA oligo substrate labeled with fluorescein and a quencher at its end. When cleaved by an RNase, the substrate fluoresces green (490 nm excitation and 520 nm emission) and can be measured by a fluorometer. RNase activity was performed in black flat-bottom 96-well plates (Invitrogen) at 37°C. Fluorogenic RNA substrate (10 pmol) was incubated with rLinCas2C, its mutant variants, and LinCas2C_Lai (25 µM) in a total of 100 μl reaction buffer (25 mM Tris-Cl pH 8.0 and 100 mM KCl). Fluorescence was measured at every 5 min interval till 60 min using the Infinite M200Pro plate reader (Tecan).

### Crystallization, data collection, and structure determination

The purified protein (rLinCas2C, 5 mg/ml) was screened for initial crystal hits using crystallization conditions available from Hampton Research utilizing the hanging-drop vapor-diffusion method at 4°C. Diffraction quality crystals of rLinCas2C were obtained in 0.2 M sodium citrate tribasic dihydrate pH 5.6, 5% 2-propanol, 20% polyethylene glycol (PEG) 4,000 and 0.2% low melting agarose (LMA). X-ray intensity diffraction data were collected at −173°C using the home source Rigaku MicroMax-007 HF diffractometer (operated at 40 kV and 30 mA) and R-Axis IV++ imaging-plate detector available at the central instrument facility (CIF) of the Indian Institute of Technology Guwahati, India. The crystal to detector distance was maintained at 170 mm. The diffraction data were processed and scaled using the programs iMosflm (Battye et al., 2011) and Aimless (Evans and Murshudov, 2013) embedded in the CCP4 package (Winn et al., 2011). The intensities were converted to structure factors using the module ctruncate available in the CCP4 package. Summary for X-ray intensity data collection and processing statistics are provided in Table 1. Initial phases of the protein rLinCas2C were determined employing the molecular replacement method using the crystal structure of SpyCas2 (PDB id: 4QR0) from *Streptococcus pyogenes* having a sequence identity (query coverage) of 45 (98)% as a search model using the program Phaser (McCoy et al., 2007). To calculate the R_free_, 5% of the total reflections were kept aside as a test data set (Brünger, 1992). The atomic model building and iterative cycles of structural parameters refinement were carried out using Coot (Emsley et al., 2010) and Refmac5 (Murshudov et al., 2011), respectively. The structural quality of the final refined model was validated using programs PROCHECK (Laskowski et al., 1993) and MolProbity (Chen et al., 2010). As the final refined model did not contain a metal (Mg^2+^) ion in its active site required for its activity, crystallization of the protein incubated with MgCl_2_ was attempted. However, a diffractive crystal could not be obtained. The details of the structure refinement and validation of the final structure models are provided in Table 1. The three-dimensional atomic coordinates of the protein LinCas2 have been deposited in the RCSB Protein Data Bank (PDB id: 7F84) (Berman et al., 2000).

**TABLE 1:** Data collection and refinement statistics of rLinCas2C. The values in parenthesis are for the last resolution shell.

Parameters	rLinCas2C
Wavelength (Å)	1.5418
Temperature (K)	100
Space group	*I*422
Unit-cell parameters (Å, °)	*a* = *b* = 103.16, *c* = 97.30, *α* = *β* = *γ* = 90.00
Resolution (Å)	72.94–2.60 (2.72–2.60)
No. of observed reflections	140,737 (17,109)
No. of unique reflections	8,390 (1,011)
Mn(I) CC (1/2)	0.998 (0.959)
Completeness (%)	100 (100)
V_M_ (Å^3^ Da^−1^)	3.19
Solvent content (%)	61.44
Mosaicity (°)	0.60
Mean I/σ(I)	12.3 (4.8)
R_merge_ ^a^ (%)	15.8 (50.4)
R_pim_ (%)	5.6 (17.8)
R_meas_ (%)	16.8 (53.5)
Multiplicity	16.8 (16.9)
R_work_/R_free_ (%)	20.87/26.96
Protein model	
No. of subunits in ASU	2
Protein atoms	1,428
Water molecules	51
Other molecules	GOL
Deviation from ideal geometry	
Bond length (Å)	0.013
Bond angles (°)	1.869
Average *B*-factor (Å^2^)	
Protein atoms	27.39
Water molecules	33.52
Other molecules	63.98
Ramachandran plot (%)	
Favored	96.53
Allowed	3.47
PDB id	**7F84**

a *R*_merge_ = ∑_*hkl*_ ∑_*i*_|*I*_*i*_(*hkl*)–⟨*I* (*hkl*)|/∑_*hkl*_∑_*i*_I_*i*_(*hkl*), where *I* (*hkl*) is the intensity of reflection *hkl, ∑hkl* is the sum overall reflections and ∑*i* is the sum over *i* measurements of reflection *hkl*, GOL:glycerol.

## Results

### *CRISPR-Cas I-C locus in the genome of L. interrogans* serovar Copenhageni *and L. interrogans* serovar Lai

**FIGURE 1:**
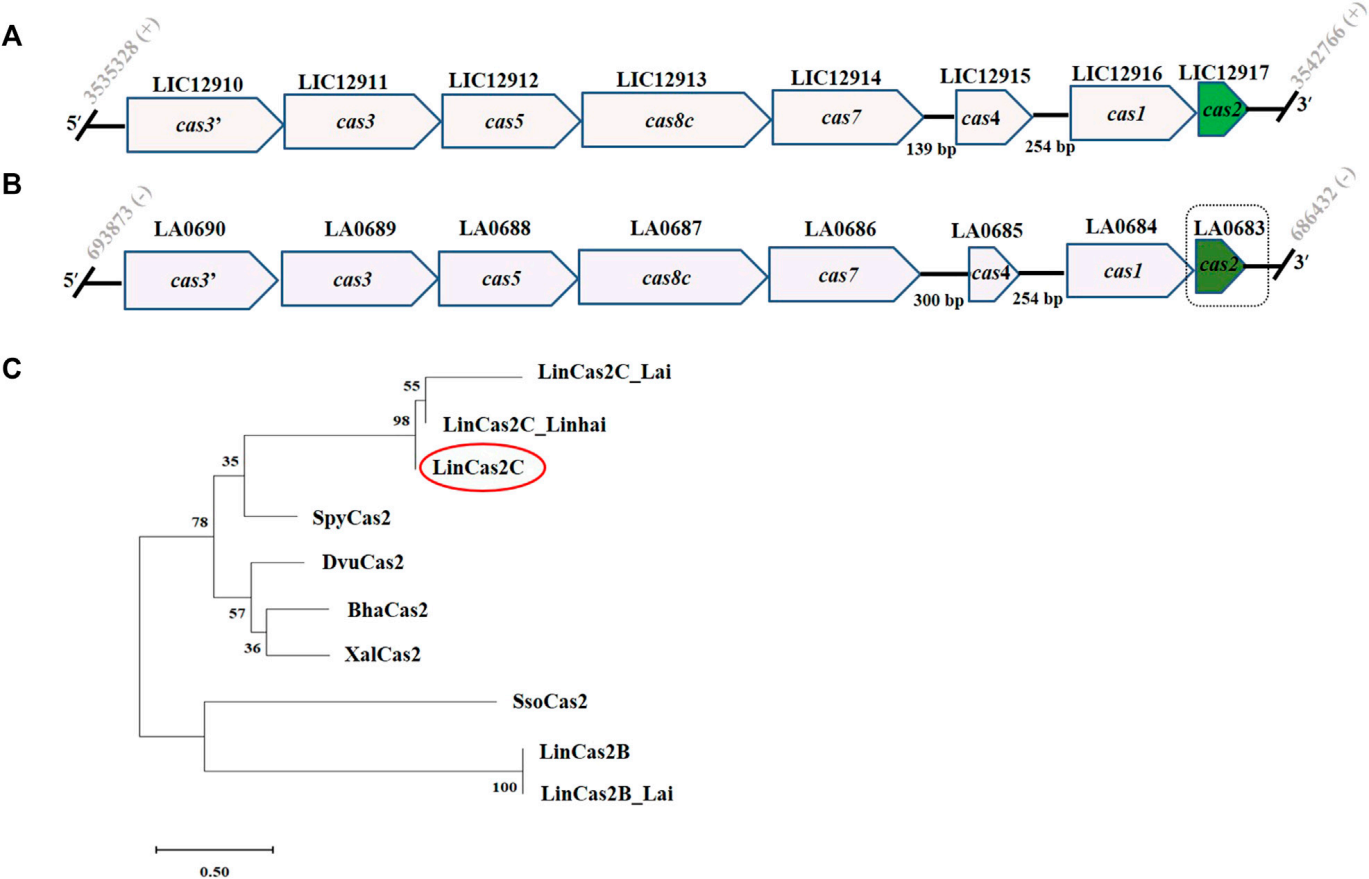
CRISPR-Cas I-C locus of *L. interrogans* and molecular phylogeny of Cas2C orthologs. **(A,B)** Schematic representation of the architecture of CRISPR-Cas I-C of serovar Copenhageni and Lai, respectively. **(C)** Phylogenetic analysis of Cas2 orthologs generated by way of the maximum likelihood algorithm. *L. interrogans* serovar Copenhageni Cas2B and Cas2C are represented as LinCas2B and LinCas2C, respectively. Similarly, in parenthesis, Cas2C of *L. interrogans* serovar Lai (LinCas2C_Lai), *Streptococcus pyogenes* (SpyCas2; Q99YS8), *Xanthomonas albilineans* (XalCas2; D2UG58), *Bacillus halodurans* (Bha_Cas2; Q9KFX8), *Desulfovibrio vulgaris* (DvuCas2; Q72WF4) and *Sulfolobus solfataricus* (SsoCas2; Q97YC2) are shown. + and − sign represents sense and anti-sense strands, respectively.

Based on the CRISPROne program (Zhang and Ye, 2017) and the data retrieved from the earlier report (Makarova et al., 2015), the CRISPR-Cas I-C locus (nucleotides coordinate 3535328–3542766) containing the *cas2c* (ORF id: *LIC12917*) gene of size 273 bp in *L. interrogans* serovar Copenhageni is illustrated (Figure 1A). In a similar *in silico* approach of genome analysis in *L. interrogans* serovar Lai, another well-studied pathogenic spirochete, the *cas2c* (ORF id: *LA0683*) gene in the CRISPR-Cas I-C locus (nucleotides coordinate 686432–693873), appeared to be absent (Xiao et al., 2019). Conversely, the NCBI genome database of *Leptospira* predicts the *cas2c* (*LA0683*) gene to be of 272 bp size with a natural deletion of one nucleotide (adenine^108^) that may result in partial ORF translation. Thus, Xiao and co-workers (Xiao et al., 2019) reported that in *L. interrogans* serovars Lai genome, *cas2c* (*LA0683*) might encode for truncated (58 amino acids and frameshift after 35th residue) and inactive LinCas2C_Lai (Xiao et al., 2019). We, thus, recount the *LA0683* partial reading frame (177 of 272 bp) in the CRISPR I-C locus of *L. interrogans* serovar Lai (Figure 1B), and all other *cas* genes are described with the CRISPR-Cas I-C locus of serovar Copenhageni (Figure 1A). Unlike the CRISPR-Cas I-B locus, the CRISPR-Cas I-C in *Leptospira* lacks the CRISPR array essential for imparting RNA-mediated interference of foreign nucleic acids. CRISPR-Cas I-C locus in the absence of array, the role of Cas2 in CRISPR biology is questionable. Phylogenetic analysis was performed to explore the evolutionary relationship of LinCas2C with the selected Cas2 homologs (Figure 1C). The lineage of LinCas2B appears to be closer to SsoCas2, whereas LinCas2C is closer to SpyCas2. However, the lineage of LinCas2C among Cas2 of *Leptospira* was closely related to LinCas2C_Linhai and LinCas2C_Lai (Figure 1C). LinCas2B is a well-studied Cas protein of *Leptospira* from our group (Dixit et al., 2016); however, LinCas2B and LinCas2C proteins formed separate clades in the phylogenetic tree analysis. The phylogenetic study encouraged us to address if the LinCas2C nuclease property is different from LinCas2B. To date, independent research groups have ascertained the *L. interrogans cas2* genes of CRISPR-Cas to be transcriptionally active in different serovars*,* and the characterization of LinCas2B and LinCas2C_Linhai demonstrated to have metal-dependent DNase activity (Dixit et al., 2016; Xiao et al., 2019). Nevertheless, there is a gap in understanding the role of LinCas2C found at subtype I-C, which lacks the essential array element.

### Recombinant LinCas2C and LinCas2C_Lai nuclease activity on double-stranded DNA

The Cas proteins are known to possess nuclease activity. During the adaptation phase of the CRISPR-Cas immunity, the Cas1-Cas2 heterohexameric complex executes its nuclease activity for new spacer integration (Lee et al., 2019). The genes *cas2c* (*LIC12917*; 273 bp) and *cas2c_*Lai (*LA0683*; 272 bp) were cloned, and the recombinant proteins (rLinCas2C and rLinCas2C_Lai) were purified using Ni-NTA affinity chromatography to investigate the nuclease activity (Supplementary Figure S1A). Due to natural frameshift mutation, rLinCas2C_Lai was purified in a truncated form (8.6 kDa), lacking the C-terminal region essential for dimer formation. To our dismay, rLinCas2C_Lai, in addition to a monomeric state (12 kDa), could self-assemble to a trimeric (34 kDa) state instead of a dimer during size-exclusion chromatography (SEC) (Supplementary Figure S1B). On the other hand, rLinCas2C (LIC12917) self-assembled in the dimeric (28 kDa) and monomeric (15 kDa) state when resolved through SEC (Supplementary Figure S1B). Specific polyclonal antibodies raised in mice against rLinCas2C and rLinCas2B did not cross-react with each other (Supplementary Figure S1C). Nevertheless, anti-LinCas2C could cross-react with rLinCas2C_Lai (Supplementary Figure S1D) and agrees with the phylogenetic study shown in Figure 1C. In addition, the monomeric and dimeric LinCas2C native expression could also be detected in *L. interrogans* serovar Copenhageni lysate (Supplementary Figure S1E).

**FIGURE 2:**
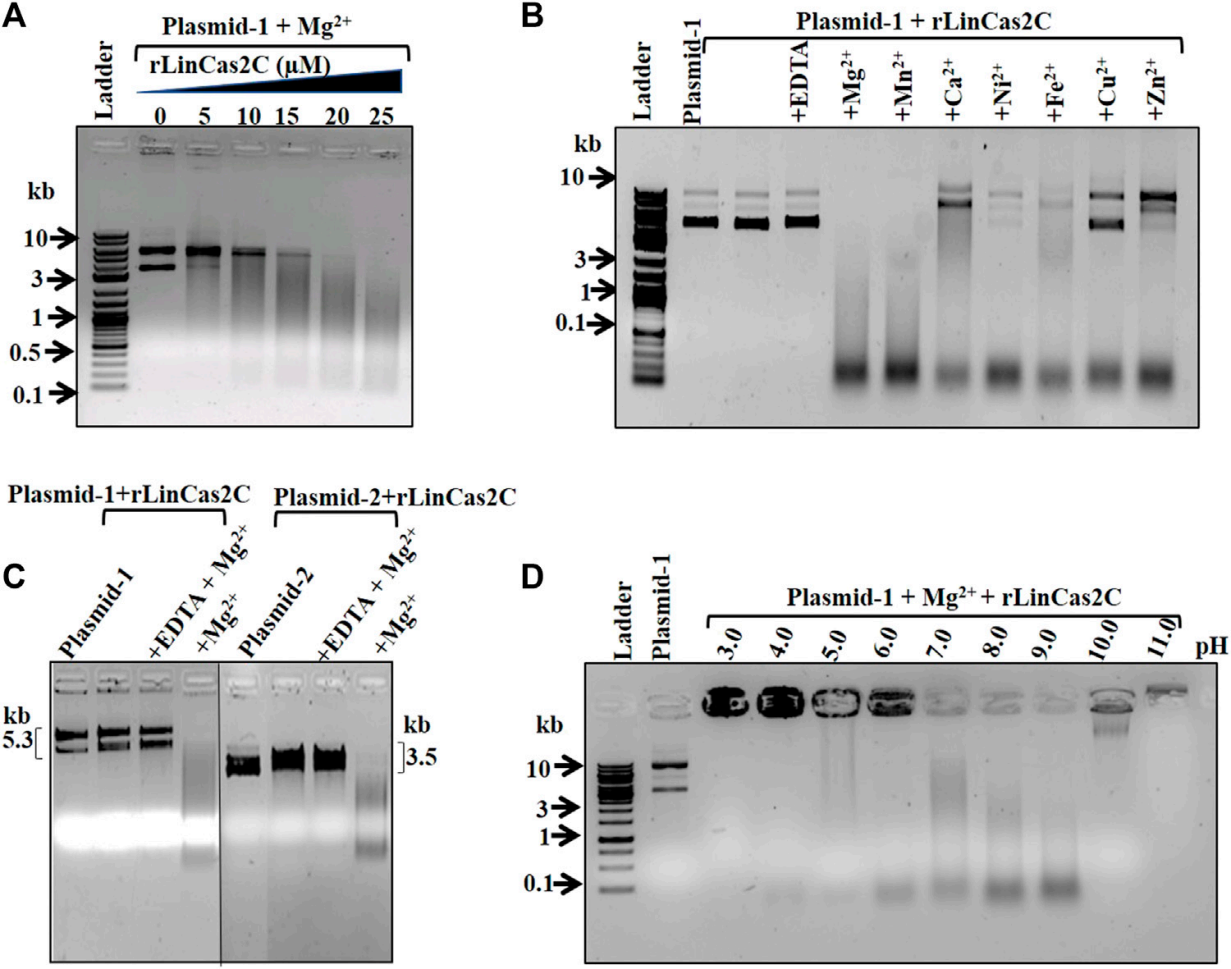
DNase activity of rLinCas2C on plasmid DNA. DNase activity reaction was carried out at 37°C for an hour. **(A)** Concentration-dependent DNase activity of recombinant rLinCas2C on plasmid-1 substrate (5.3 kb pET28a vector, 0.5 µg) in the presence of Mg^2+^ ion. Complete cleavage of the substrate was observed using rLinCas2C at 25 µM. **(B)** DNase activity of rLinCas2C in the presence of different divalent metal ions on plasmid exemplifies its optimum activity in Mg^2+^ and Mn^2+^ ions. **(C)** The substrate specificity of rLinCas2C on two different plasmid substrates. Substrate plasmid-1 and plasmid-2 (3.5 kb pTZ57 R/T vector, 0.5 µg) were employed for DNase activity. **(D)** DNase activity of rLinCas2C at different pH. The optimum activity was observed at pH 8.0 and 9.0. DNA ladder: 2 log DNA ladder (NEB). rLinCas2C: 25 μM, EDTA: 2.5 mM, Mg^2+^ and others divalent metal: 2.5 mM. Reaction products were analyzed on 2% agarose gel.

The purified Cas2C (rLinCas2C and rLinCas2C_Lai) was used to investigate the nuclease activity on various DNA substrates. We excluded LinCas2B_Lai in our analysis as it had 100% sequence similarity to the well-studied LinCas2B. In a nuclease assay, increasing concentrations (5–25 µM) of each rLinCas2C and rLinCas2C_Lai were taken to optimize the cleavage of the dsDNA (plasmid DNA; 0.5 µg). Around 25 µM of each rLinCas2C and rLinCas2C_Lai could completely cleave the DNA (0.5 µg) in an hour at 37°C (Figure 2A; Supplementary Figure S2A). The DNA cleavage assay of rLinCas2C and rLinCas2C_Lai on circular dsDNA suggested that both Cas2C are endodeoxyribonucleases.

For studying metallonucleases, substituting metal ions is a common practice to understand their role in the nuclease activity (Dixit et al., 2016; Dixit et al., 2021). Mg^2+^ ion was substituted with other divalent metal ions (Mn^2+^, Ca^2+^, Ni^2+^, Fe^2+^, Cu^2+^, and Zn^2+^) to explore their (rLinCas2C and rLinCas2C_Lai) preference for DNase activity. Both the nucleases (rLinCas2C and rLinCas2C_Lai) displayed higher affinity towards Mg^2+^, followed by Mn^2+^ and Fe^2+^ as a cofactor for its DNase activity. LinCas2B and BhaCas2 also preferred Mg^2+^ over other metal ions (Nam et al., 2012; Dixit et al., 2016) and are in coalition with the cofactor preference assay. In the presence of Ca^2+^, Cu^2+^, and Zn^2+^, a curtailed or no DNA cleavage activity was exhibited by both the nucleases (Figure 2B; Supplementary Figure S2B). A shift in DNA mobility was also detected during agarose gel electrophoresis in the presence of cofactors Ca^2+^, Cu^2+^, and Zn^2+^. Such a shift could be due to the retainment of the DNA binding property of LinCas2C.

The nuclease assays of LinCas2B and LinCas2C_Linhai under *in vitro* conditions demonstrated that Cas2 proteins exhibit divalent metal and pH-dependent nuclease activities, where the substrate preferences fluctuated incredibly (Dixit et al., 2016; Xiao et al., 2019). Thus, it was intriguing to address whether the rLinCas2C nuclease activity is dependent on the nucleotide sequence. The DNase activity of rLinCas2C was conducted on two substrates (circular dsDNA plasmid). Both the nucleases (rLinCas2C and rLinCas2C_Lai) exhibited DNase activity non-specifically similar to that of LinCas2B (Dixit et al., 2016) and LinCas2C_Linhai (Xiao et al., 2019). The divalent metal ions were prerequisites for DNase activity in rLinCas2C, as the addition of EDTA completely abolished the plasmid degradation (Figure 2C). The rLinCas2C exhibited optimum DNase activity in the pH range of 7.0 and 9.0. Nuclease activity gets reduced at pH 10.0 to 11.0 and exhibits a moderate affinity for DNA (Figure 2D). The pH-dependent DNase activity of rLinCas2C agreed with that of LinCas2B (Dixit et al., 2016). Similarly, it is proposed that at the optimum pH, Cas2 (BhaCas2) attains a metal-bound catalytically active conformation (Nam et al., 2012).

### Recombinant LinCas2C nuclease activity on single-stranded DNA and RNA

**FIGURE 3:**
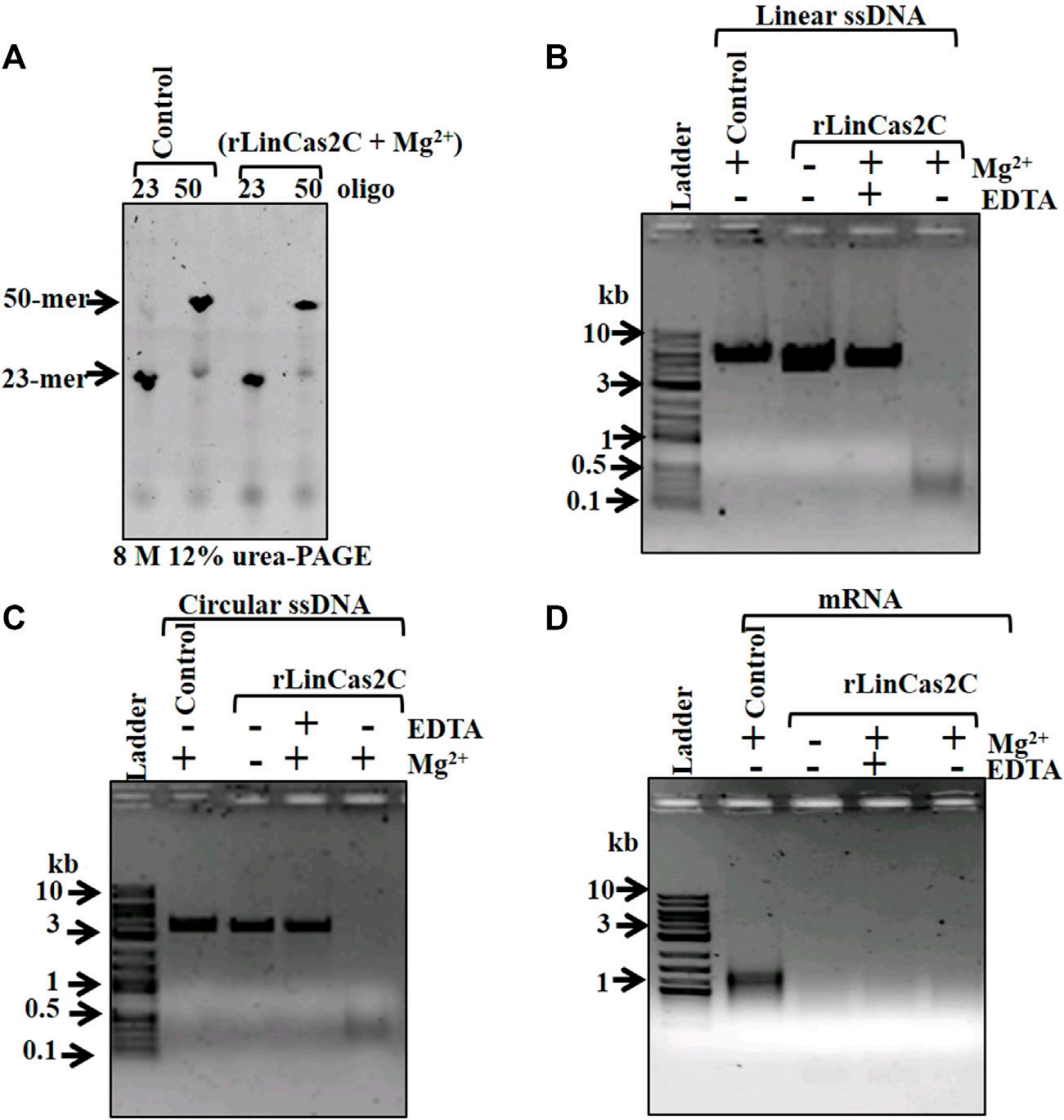
Nuclease activity of rLinCas2C on single-stranded DNA and RNA. The nuclease activity reaction was carried out at 37°C for an hour. **(A)** DNase activity of rLinCas2C on synthesized single-stranded linear DNA (oligo-1: 23-mer 0.4 µM, oligo-2: 50-mer 0.4 µM). The nuclease reaction product was analyzed on 8 M 15% urea-PAGE. **(B)** DNase activity of rLinCas2C on linear single-stranded DNA (0.5 µg of 6.4 kb M13mp18). Complete degradation of linear single-stranded was observed in the presence of Mg^2+^ ions. **(C)** DNase activity of rLinCas2C on circular single-stranded DNA (3.6 kb ϕx174, 0.5 µg). Complete degradation of circular single-stranded was observed in the presence of Mg^2+^ ion. **(D)** RNase activity of rLinCas2C on *luciferase* mRNA (0.5 µg). Complete degradation of RNA was observed even in the absence of Mg^2+^ ions. DNA ladder: 2 log DNA ladder (NEB). rLinCas2C: 25 µM and Mg^2+^: 2.5 mM. The nuclease reaction products shown in Figure **(B)**, **(C)**, and **(D)** were analyzed on 2% agarose gel.

Since rLinCas2C degraded dsDNA, it was intriguing to evaluate its activity on ssDNA and ssRNA. In a previous study, LinCas2B and BhaCas2 were inert toward short DNA oligos (28–32-mer) (Nam et al., 2012; Dixit et al., 2016). In agreement, rLinCas2C could not cleave short DNA oligos (23- and 50-mer) in the presence of a cofactor (Figure 3A). The DNase activity of rLinCas2C and rLinCas2C_Lai on the viral ssDNA (linear M13mp18 and circular Фx174) demonstrated cleavage in the presence of divalent metal ion (Figures 3B,C; Supplementary Figures S2C,D). On the same line, LinCas2B, in the presence of a cofactor, also cleaves viral ssDNA (Dixit et al., 2016). In addition, rLinCas2C and rLinCas2C_Lai exhibited cleavage of mRNA transcripts of *luciferase* gene independent of divalent metal ions (Figure 3D; Supplementary Figure S2E).

### Overall structure of rLinCas2C

**FIGURE 4:**
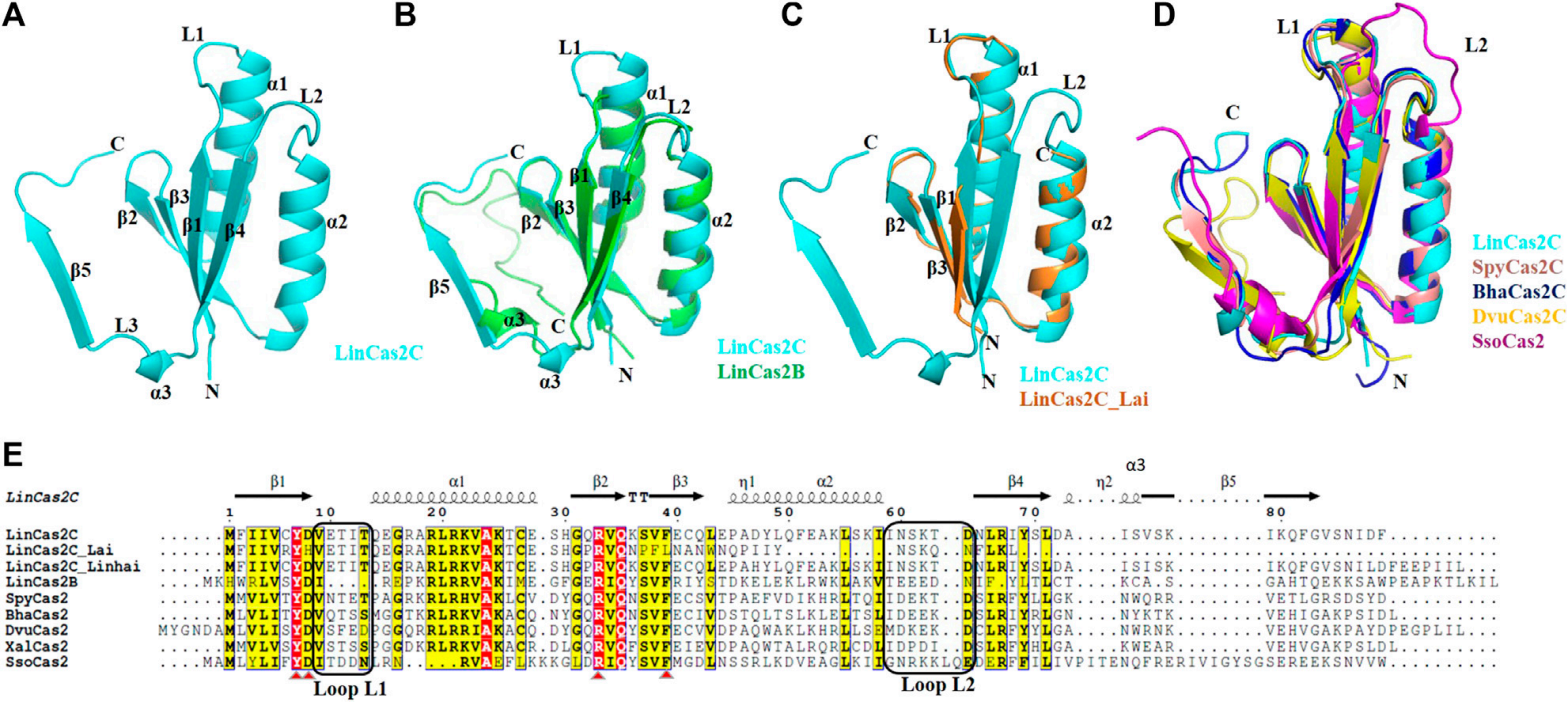
The crystal structure of rLinCas2C and its correlation with various orthologs. **(A)** The crystal structure of rLinCas2C is represented as a cartoon model. All the secondary structural elements, along with the N-and C-termini, are labeled for clarity. In order to map the putative substrate-binding loop (L1 and L2), rLinCas2C structure was correlated with LinCas2B; rmsd: 0.8 **(B)** LinCas2C_Lai; rmsd: 1.0 **(C)**, SpyCas2; rmsd: 0.9, BhaCas2; rmsd: 0.6, DvuCas2; rmsd: 0.9, SsoCas2; rmsd: 1.4 Å **(D)**. **(E)** Multiple sequence alignment of LinCas2C with its orthologs. Two putative substrate-binding loops, L1 (DNA) and L2 (RNA), and secondary structure elements, are labeled. The secondary structural elements on top of the alignment are given according to the rLinCas2C. XalCas2: *Xanthomonas albilineans* (D2UG58), BhaCas2: *Bacillus halodurans* (Q9KFX8), DvuCas2: *Desulfovibrio vulgaris* (Q72WF4), SsoCas2: *Sulfolobus solfataricus* (Q97YC2), SpyCas2: *Streptococcus pyogenes* (Q99YS8). Loop L1 and L2 are marked with rectangles. Red triangles highlight conserved residues.

The crystal structure of rLinCas2C encloses the signature N-terminal ferredoxin domain (βαββαβ). LinCas2C crystal is composed of a total of three α-helices (α1–α3) and five anti-parallel β-strands (β1–β5) (Figure 4A), as described before for the Cas2 orthologs enlisted in Table 2. The solvent-accessible surface area and Gibbs free energy of monomeric rLinCas2C were 6,563.8 Å^2^ and −73 kcal/mol, respectively. There are two loops named loop L1 and L2 connecting β1-α1 and α2-β4, respectively, found in all Cas2 orthologs. Loops L1 and L2 are speculated to recognize DNA and RNA substrates, respectively (Beloglazova et al., 2008). Structure superimposition of rLinCas2C over modeled rLinCas2B shows a shorter DNA binding loop L1 while the RNA binding loop L2 was comparable in size (Figure 4B). The modeled three-dimensional structure of rLinCas2C_Lai disclosed the presence of two α-helices (α1 and α2) and three anti-parallel β-strands (β1-β3). However, β4 and β5, the two β-strands at the C-terminus, are missing compared to rLinCas2C (Supplementary Figure S3A). Structural superimposition of rLinCas2C_Lai revealed identical DNA binding loop L1 as rLinCas2; however, shorter loop L2. In LinCas2C_Lai, amino acid residues imparting loop L2 were intact even after frameshift mutation (Figure 4C). Intact loop L2 may be the possible reason behind rLinCas2C_Lai displaying activity despite expressing the truncated protein.

**TABLE 2:** List of structural homologs of LinCas2C.

Protein	PDB id	Rmsd (Å)	Z-Score	References
BhaCas2	4ES1	1.2	14.7	Nam et al. (2012)
SpyCas2	4QR2	1.2	14.6	Ka et al. (2014)
XalCas2	5H1O	1.6	13.8	Ka et al. (2017)
TthCas2	1ZPW	1.8	11.3	Seto et al. (2003)
DvuCas2	3OQ2	1.9	13.4	Samai et al. (2010)
PhoCas2	6K2E	1.9	10.4	—
TonCas2	5G4D	1.9	10.5	Jung et al. (2016)
PfuCas2	2I0X	2.0	10.1	—
TdeCas2	6JHZ	2.2	9.3	—
SpyCas2	5ZYF	2.5	9.7	Ka et al. (2014)
SsoCas2	2I8E	2.5	9.3	Beloglazova et al. (2008)
EcoCas2	4MAK	—	—	—

Structural homology search based on scores of selected parameters [Z-score and root mean square deviation (rmsd)] of the web server DALI (Holm, 2020) revealed the closest homologs of rLinCas2C to be BhaCas2 and SpyCas2 (Table 2).

In addition, the crystal structure of rLinCas2C was superimposed with the structures of Cas2 orthologs (SpyCas2C, BhaCas2C, DvuCas2C, and SsoCas2) and the putative DNA (L1) and RNA (L2) binding loops were compared (Figure 4D). SpyCas2C, BhaCas2C, and DvuCas2 have identical L1 and L2 loop sizes to rLinCas2C. Similarly, to understand the rLinCas2C_Lai divergence in the putative substrate-binding loop, its modeled structure (Supplementary Figure S3A and PDB file in supplementary information) was superimposed with the SpyCas2, BhaCas2, DvuCas2, and SsoCas2 (Supplementary Figures S3B–S3E). The putative loop L1 size of rLinCas2C_Lai aligns with SpyCas2, BhaCas2, and DvuCas2 but not SsoCas2. However, the putative loop L2 of rLinCas2C_Lai was smaller than its orthologs (Supplementary Figures S3B–S3E).

A multiple sequence alignment of LinCas2C with its orthologs also displays the variation in the residues responsible for constituting the loop L1 and L2 (Figure 4E). Notably, LinCas2C_Lai shares a 31% amino acids sequence dissimilarity with LinCas2C, where few conserved residues (His8, Pro37, Phe38, Leu39, Trp44, Asn54, and Lys57) differ from their corresponding residues in LinCas2C (Asp8, Ser37, Val38, Phe39, Leu44, Asp64, and Arg67) (Figure 4E). Another Cas2C paralog of serovar Linhai (LinCas2C_Linhai) shared 3% dissimilarity at N- (Pro32 and His47) and C-terminal region (Ile78, Glu91, Glu92, Pro93, Ile94, Ile95, and Leu96) to LinCas2C (Figure 4E).

**FIGURE 5:**
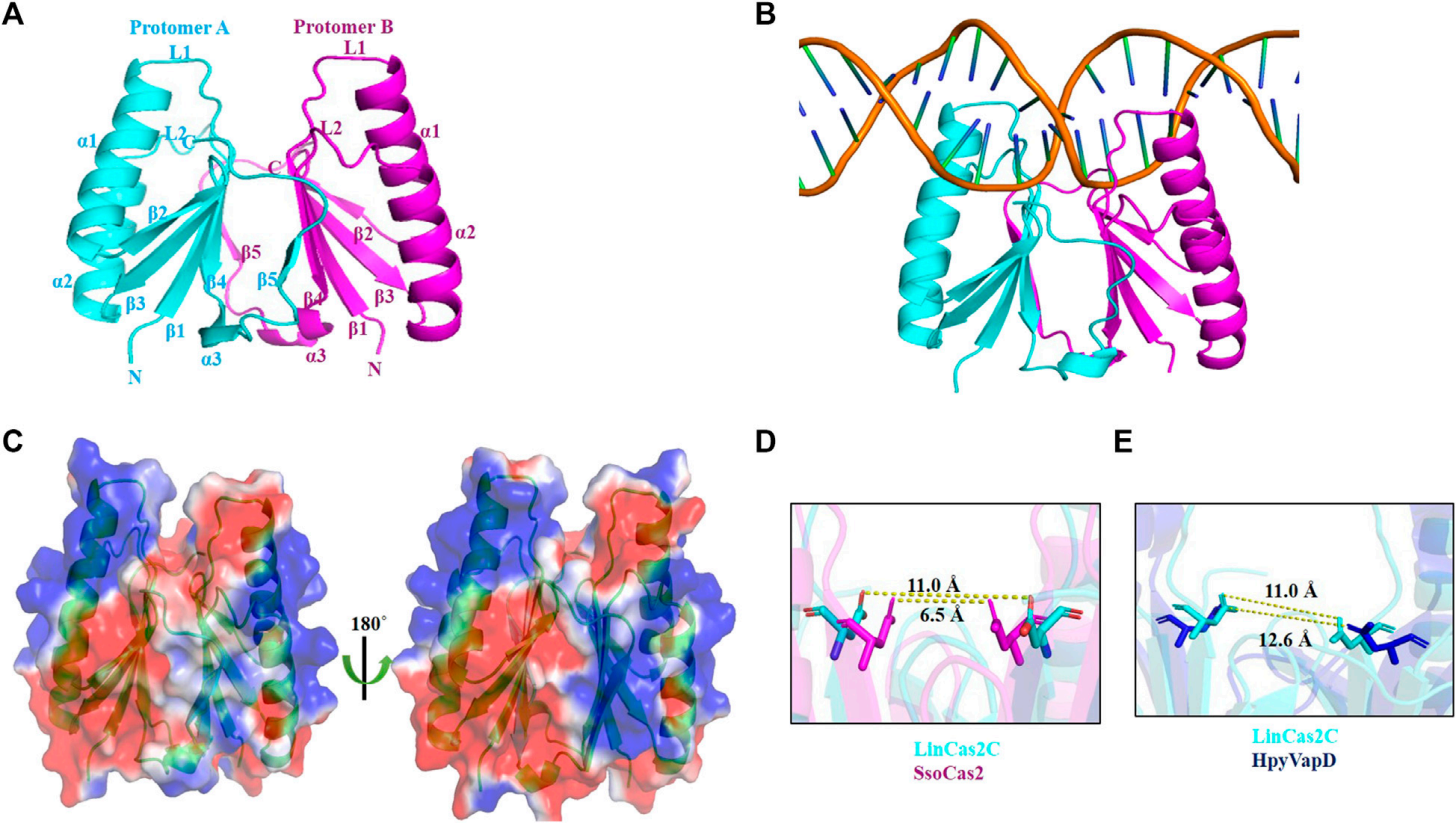
The dimeric interface of rLinCas2C crystal structure. **(A)** The crystal structure shows a dimeric form of rLinCas2C, where one protomer is shown in cyan and another in magenta. **(B)** Putative DNA binding region on LinCas2C. LinCas2C bound DNA model using the template of *Synechocystis* Cas1-Cas2/prespacer binary complex structure (PDB id:7CR6). **(C)** Surface electrostatic potential map of LinCas2C. The positive and negative charges are blue and red, respectively (scale -1 kcal/mol to +1 kcal/mol for red and blue, respectively). **(D)** Comparison of the distance between Asp residues side chain of the two protomers of LinCas2C and SsoCas2 and **(E)** LinCas2C-HpyVapD.

The asymmetric unit of the rLinCas2C crystal contains two protein subunits forming a dimer (Figure 5A) and agrees with the crystal structure of Cas2 orthologs enlisted in Table 2 (Beloglazova et al., 2008; Samai et al., 2010; Ka et al., 2014). In SpyCas2, upon dimerization, the surface area buried is 2,793–2,856 Å^2^, forming 29–32 hydrogen bonds between the two protomers (Ka et al., 2014). In rLinCas2C, upon dimerization, the buried surface area is 3,430 Å^2^, identified by PDBePISA (Velankar et al., 2010). A total of 33 hydrogen bonds were formed between two protomers of LinCas2C as analyzed by Coot (Emsley et al., 2010). The rLinCas2C dimer demonstrates that the β5 strand (6 residues) of one protomer bridges with the β4 strand (5 residues) of another protomer by 8–10 hydrogen bonds and several other residues enlisted in Supplementary Table S2. Interestingly, residues (Asp8, Asp64, Leu66, and Arg67) present at the dimeric interface were conserved among LinCas2C orthologs. To our dismay, rLinCas2C_Lai lacks β4 and β5 strands but still exhibits a trimeric state in solution. The trimeric structure of modeled LinCas2C_Lai was predicted by generating symmetry mate. Trimeric structural analysis revealed that Arg6, His8, Gln35, and Asn36 of one protomer interact with Gln35, Asn36, Arg6, and His8 of the second protomer. Arg17 of the third protomer interacts with Ser29 of the second protomer at a distance of ≤3.5 Å (data not shown). Such interaction may be the probable reason for the self-assembly of rLinCas2C_Lai as a trimer in solution.

The rLinCas2C DNA-nuclease interface demonstrates that the two complementary strands of DNA are cleaved by each protomer of the nuclease (Figure 5B). The rLinCas2C residues interacting with DNA are primarily from the loop L1, L2, and α1 regions (Supplementary Table S3). In agreement, the heterocomplex Cas1-Cas2-dsDNA structure of *E. coli* showed that the residues constituting the L1 loop of Cas2 interact with dsDNA (Nunez et al., 2015). The mapping of the surface electrostatic potential of rLinCas2C demonstrated the presence of a positive charge at the putative nucleic acid substrate-binding loop (L1 and L2) and the α1 region (Figure 5C). The mapped amino acid residues of rLinCas2C interacting with dsDNA are shown in Supplementary Table S3 and the PDB file of the supplementary information. Among all the enlisted interacting residues, seven amino acid residues were positively charged (Agr17, Arg21, Arg33, Lys36, and Lys62). The crystal structure of rLinCas2C (dimeric form) indicates it is in a catalytically inactive conformational state as the distance between the conserved Asp8 residue of each protomer is 11.0 Å (as opposed to 6.5 Å in SsoCas2) (Figure 5D). The distance of 11.0 Å seems too far to coordinate a single Mg^2+^ ion of the protein. Similarly, the protomers of SpyCas2 (11.4 Å), BhaCas2 (10.6 Å), DvuCas2 (15.4 Å), and HpyVapD (12.6 Å) measured uneven distance between the conserved equivalent aspartate residue (Figure 5E) (Beloglazova et al., 2008; Samai et al., 2010; Nam et al., 2012; Ka et al., 2014; Bertelsen et al., 2021).

### Recombinant LinCas2C mutants and their activity

**FIGURE 6:**
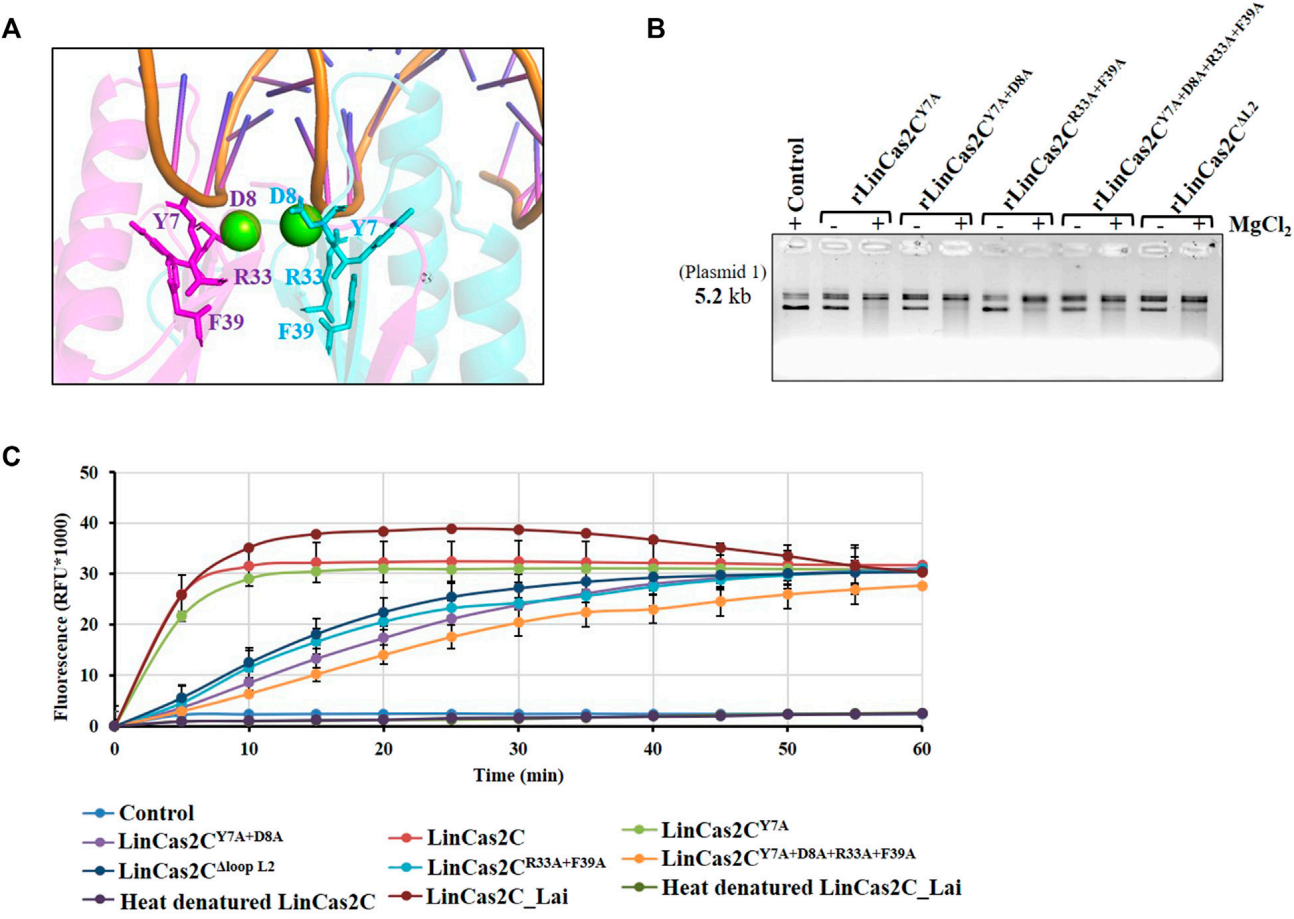
Nuclease activity of rLinCas2C mutant variants. **(A)** Proposed model of LinCas2C with Mg^2+^ ion. LinCas2C amino acid residues interacting with metal ions and putative active site are shown in stick form. **(B)** Nuclease activity of rLinCas2C mutants was evaluated on plasmid-1 (0.5 µg) in the presence and absence of divalent metal ion. **(C)** RNase activity of rLinCas2C or its mutant variants and the rLinCas2C_Lai was quantified using fluorescently labeled RNA substrate. A fluorescent RNA substrate (10 pmol) was incubated with rLinCas2C or its mutants (25 µM) at 37°C, and fluorescence was recorded at 5 min intervals for 1 hour.

A multiple sequence alignment of LinCas2C with its orthologs illustrated similarity with SpyCas2 (sequence similarity: 45% and query coverage: 98%), XabCas2 of *Xanthomonas albilineans* (41 and 100%), BhaCas2 (39 and 100%), DvuCas2 (37 and 100%), and SsoCas2 (30 and 68%). Several conserved residues (Tyr7, Asp8, Ala24, Arg33, Gln35, Leu55, and Leu71) and motifs (RVQ and SVF) in LinCas2C were identified (Figure 4E). We have shown previously that mutation of Asp10 of LinCas2B abolished its DNase activity but not its RNase activity (Dixit et al., 2016). Thus, in this study, an additional site-directed mutation was performed in rLinCas2C at one or more sites predisposed to nuclease activity, and the purified recombinant protein was obtained for its characterization (Supplementary Figure S1A). A model of LinCas2C with metal-ion was proposed to map the metal-ion binding residues. Tyr7 and Asp8 were found to be putative metal-binding residues of LinCas2C (Figure 6A). Two other residues, Arg33 and Phe39, were found close to metal-binding residues and putative active site groove [purposed by Yakunin and co-workers (Beloglazova et al., 2008)], and were also found to be conserved among Cas2 homologs (Figure 6A).

In SsoCas2, the residues Arg31 and Phe37 have been essential for nuclease activity (Beloglazova et al., 2008). The rLinCas2C with single (rLinCas2C^Y7A^) and double (rLinCas2C^Y7A+D8A^) mutants demonstrated reduced DNase activity, where a change in conformation of plasmid was evident due to a partial nick in DNA (Figure 6B). In agreement, in this study, additional mutation of Arg33 and Phe39 in rLinCasC (rLinCas2C^R33A+F39A^ and rLinCas2C^Y7A+D8A+R33A+F39A^) exhibited complete abolition in DNase activity (Figure 6B). Yakunin and co-workers speculated that DNA and RNA substrate might interact with Cas2 loop L1 (β1-α1) and L2 (α2-β4), respectively (Beloglazova et al., 2008). Hence, to explore the role of loop L2 in rLinCas2, a mutant construct with L2 deletion (rLinCas2C^ΔL2^) was generated to analyze the DNase and RNase activity. To our surprise, rLinCas2C^ΔL2^ displayed a complete loss of DNase activity (Figure 6B). The DNase assay with rLinCas2C^ΔL2^ conflicted with an earlier report (Beloglazova et al., 2008), where the loop L1 was speculated for DNA substrate recognition. To address this inconsistency, rLinCas2C was docked with random DNA. Analysis of the docking study of DNA-LinCas2C suggests that DNA may interact with rLinCas2C at multiple sites, including the residues (Asp60, Lys62, Thr63, and Asp64) that constitute the loop L2 (Supplementary Figure S4).

A kit-based fluorogenic RNA substrate was employed to quantify the RNase activity of rLinCas2C and compare it with its mutants or rLinCas2C_Lai. The mutant rLinCas2C^Y7A^ exhibited RNase activity very similar to rLinCas2C, while the activity of other mutants (rLinCas2C^Y7A+D8A^, rLinCas2C^R33A+F39A^, rLinCas2C^Y7A+D8A+R33A+F39A^, and rLinCas2C^ΔL2^) reduced moderately (Figure 6C). The RNase activity of mutant rLinCas2C^Y7A+D8A+R33A+F39A^ was affected most adversely; however, none of the mutants demonstrated complete abolition in RNase activity. The RNase activity of rLinCas2C and rLinCas2C_Lai were abolished after heat denaturation, indicating protein is free of RNase contaminant, and activity is dependent on Cas2C protein conformation. The RNase assay suggested that in LinCas2, the residues involved in RNA cleavage differ from the DNA and thus has metal independency.

## Discussion

The recombinant LinCas2C and the naturally truncated LinCas2C_Lai demonstrated nuclease activity on diverse DNA substrates (circular dsDNA, linear, and circular ssDNA) in a divalent metal- and pH-dependent manner. However, these nucleases were inert toward small DNA oligos (23-50-mer). In a recent study, LinCas2C_Linhai, a Cas2C ortholog, prefers Mg^2+^ for nuclease activity (Xiao et al., 2019). In contrast, LpnCas2 of *Legionella pneumophila* and TthCas2 of *Thermus thermophilus* could demonstrate nuclease activity in the presence of Mn^2+^ (Nam et al., 2012; Gunderson et al., 2015). The DNase activity of rLinCas2C and rLinCas2C_Lai was consistent with the other reported Cas2 proteins, including BhaCas2 (Nam et al., 2012), SpyCas2 (Ka et al., 2014), XorCas2 of *Xanthomonas oryzae* (Makarova et al., 2011), and LinCas2B (Dixit et al., 2016). Consistent with LinCas2B activity, rLinCas2C was inert towards single-stranded short oligos (Dixit et al., 2016). The recombinant Cas2 nucleases (LinCas2C and LinCas2C_Lai) of the two serovars of *Leptospira* is a divalent metal-independent RNase. In contrast, Sso8090Cas2 homologs from *Sulfolobus solfataricus,* TmaCas2 of *Thermotoga maritima*, MthCas2 of *Methanobacterium thermoautotrophicum*, AfuCas2 of *Archaeoglobus fulgidus,* LpnCas2 and NeuCas2 of *Nitrosomonas europea* exhibited metal-dependent RNase activity (Beloglazova et al., 2008). Detection of nuclease activity in rLinCas2C_Lai suggests that the conserved residues at the N-terminal are more involved in nucleic acid catalysis. The metal-independent RNase activity of rLinCas2C indicates its additional role beyond CRISPR biology.

In a recent study, the virulence-associated protein D (VapD) of toxin-antitoxin systems (TA) was shown to possess a ribonuclease fold similar to Cas2 proteins (Bertelsen et al., 2021). The VapD toxins act as metal-independent nucleases that modulate gene expression by degrading specific, stable RNAs, including tRNA, rRNA, and mRNA (Goeders and Van Melderen, 2014). However, structurally VapD possesses a modified ferredoxin fold (β1α1β2-β3α2β4), where each of the two α-helices is split into two shorter helices connected by short loops, resulting in a β1α1′α1β2β3α2α2′β4 topology. In addition, various other VapD homologs (RelE, MazF, and VapC) of a toxin-antitoxin (TA) system function as RNases (Kwon et al., 2012; Bertelsen et al., 2021). Structural similarity of VapD with Cas2 fuelled the notion that the bacterial CRISPR-Cas immunity systems might have evolved from a primordial vapXD-type TA system (Makarova et al., 2012). Further, in this study, the naturally truncated rLinCas2C_Lai retains its nuclease activity like the full-length Cas2C nucleases (LinCas2C and LinCas2B). It is speculated that the RNase property of Cas2 orthologs may degrade exotic phage transcripts or inhibit translation by mRNA cleavage globally (Bertelsen et al., 2021). Cas2 proteins may utilize the intrinsic metal-independent ribonuclease activity encoded in the VapD-like fold to modulate bacterial cell growth and survival during infection (Gunderson et al., 2015).

The structural investigation of rLinCas2C demonstrates it to exist in a dimeric and apostate conformation with each subunit containing the signature ferredoxin fold. The rLinCas2C structure confirms the evolutionary conservation of the VapD/Cas2-like ribonuclease protein fold provided by Bertelsen et al. (2021). In LinCas2C, dimeric interface β4 of one protomer interacts with β5 of another protomer, similar to SsoCas2, where the β-strand (β5) of each protomer interacts with the β-sheet of the other monomer creating a two-joint, five-strand, anti-parallel β-sheets (Beloglazova et al., 2008). Also, in TonCas2, the C-terminal region of β5 from each protomer interacts with the β4 of the other molecule to form a β-sheet of five strands in both subunits (Jung et al., 2016). The structure of rLinCas2C describes the role of the catalytic aspartate in limiting conformational freedom. The distance between conserved aspartate residues of each protomer is crucial for coordinating metal-ion. In the case of LinCas2C, it was found to be 11.0 Å seems too far to coordinate a single Mg^2+^ ion. For these aspartates to bind a bridging metal, the rLinCas2C would need to undergo either a major conformational change of the β1 or ferredoxin fold region or altogether the dimer orientation. Similarly, the uneven distance between the conserved equivalent aspartate residue was observed for the protomers of SpyCas2, BhaCas2, DvuCas2, and HpyVapD. (Beloglazova et al., 2008; Samai et al., 2010; Nam et al., 2012; Ka et al., 2014; Bertelsen et al., 2021). For DNA, since divalent cations are involved in catalysis, it is also possible that one metal ion is symmetrically bound in each site.

Metal-independent RNase activity of rLinCas2C functionally corroborates with that of HpyVapD of *H. pylori* (HP0315); however, dissimilar to that of SsoCas2. In SsoCas2, the coordination between Mg^2+^ and two Asp10 residues from two dimer subunits is mandated for initiating the phosphodiester cleavage. These two Asp10 residues from SsoCas2 dimer molecules can create coordination with an Mg^2+^ ion, as the distance between the side chains from the two residues is only 6.5 Å. However, in the case of HpyVapD or rLinCas2C, the distance between the side chains of two instances of Asp7 or Asp8 are greater than 10 Å indicating its inability to coordinate the metal ion. HpyVapD showed ribonuclease activity without metal ions (Kwon et al., 2012). At this point, the exact mechanism of these two aspartate residues as a nucleophile in the absence of metal is difficult to justify. However, considering the mutational studies of HpyVapD and its comparison with Cas2, two aspartate residues (Asp7 and Asp76) have been proposed as strong candidates for the catalytic site of VapD. In metal-independent nucleases, 2′-OH of ribose makes an intramolecular nucleophilic attack on the adjacent 3′-phosphate and breaks the RNA backbone (Yang, 2011). This mechanism is usually based on acid-base catalysis, where active-site acidic and basic residues are involved (Yang, 2011).

The closest homolog SpyCas2 is a metal- and pH-dependent dsDNase and shares standard functional features with the BhaCas2 (Ka et al., 2014). Mutagenesis of SsoCas2 (SSO1404) identified six residues (Tyr9, Asp10, Arg17, Arg19, Arg31, and Phe37) important for RNase activity and suggested that Asp10 might be the principal catalytic residue (Beloglazova et al., 2008). However, in DvuCas2, neither Tyr13 nor Phe45 was disposed of a catalytic role due to its buried location (Samai et al., 2010). Two or three conserved acidic residues are critical for catalysis in most known RNases. They involve coordinating one or two metal cations, which activate a nucleophilic water molecule to hydrolyze the phosphodiester bond or stabilize the transition state in cleavage reactions (Worrall and Luisi, 2007). In LinCas2C, alanine replacement mutation of conserved residues Tyr7, Asp8, Arg33, and Phe39 and loop L2 abolishes DNase activity, whereas moderate reduction of RNase activity was evident in selected mutants. The variation in the nuclease activity of the Cas2 family has been proposed to be due to the structural difference at its catalytic site (Nam et al., 2012). There is an exciting future building from the current work on deciphering shared protein structure-function relationships between bacterial defense systems. The global inhibition of translation by mRNA cleavage may be a fundamental principle in the biological role of Cas2 proteins as reported for TA systems, including RelBE, MazEF, PemIK, and ChpBIK (Masuda et al., 1993; Gerdes et al., 2005; Zhang et al., 2005; Beloglazova et al., 2008; Zhang and Inouye, 2009). To better understand the RNA catalysis mechanism of Cas2, a structure with RNA substrate-bound is needed. Such a structure would be highly valuable and provide insights into RNase activity.

## Data Availability

The datasets presented in this study can be found in online repositories. The names of the repository/repositories and accession number(s) can be found in the article/Supplementary Material.
