# Supplementary material for: Structural and functional characterization of Cas2 of CRISPR-Cas subtype I-C lacking the CRISPR component
Source: Front Mol Biosci. 2022 Sep 12;9:988569. doi: 10.3389/fmolb.2022.988569 (PMC9510766; doi:10.3389/fmolb.2022.988569)
Supplement: Supplementary file 1 [file Table1.docx]

**Supplementary information**

**Structural and functional characterization of Cas2 of CRISPR-Cas subtype I-C lacking the CRISPR component**

Vineet Anand*, Harshini Sheeja Prabhakaran*, Prerana Gogoi, Shankar Prasad Kanaujia#, and Manish Kumar#

Department of Biosciences and Bioengineering, Indian Institute of Technology Guwahati,

Guwahati -781039, Assam, India

*Equal contribution

#corresponding authors:

Manish Kumar and Shankar Prasad Kanaujia

Department of Biosciences and Bioengineering, Indian Institute of Technology Guwahati, Guwahati-781039, Assam, India

Email: mkumar1@iitg.ac.in

Phone: +91-361-258-2230

Fax: +91-361-258-2249

**Running Title: Structure of LinCas2C and its nuclease activity**

***Materials and Methods***

***Protein overexpression and purification.*** The recombinant LinCas2C were overexpressed in *E. coli* BL21 (DE3) cells with 1 mM isopropyl β-D-1-thiogalactopyranoside (IPTG) at 37ºC for 4 h. The purification of rLinCas2C and rLinCas2C_Lai was initially carried out in native conditions. However, rLinCas2C_Lai was expressed in truncated form (8.6 kDa) and remained insoluble in native conditions. Therefore, rLinCas2C_Lai was purified using the hybrid method described before [1-3]. Purified rLinCas2C and LinCas2C_Lai was dialysed against protein storage buffer (25 mM Tris-Cl (pH 8.0), 100 mM NaCl and 10% glycerol). The yield of rLinCas2C and rLinCas2C_Lai purification was 5 mg and 40 µg per liter, respectively, and the purified proteins were stored at -20˚ till further use. Polyclonal antibody was generated against rLinCas2C as described before [4].

***Size-exclusion chromatography.*** Size-exclusion chromatography was performed using Superdex 200 increase column (GE Healthcare, catalog. no.28-9909-44) on AKTA prime plus (GE Healthcare). The column was equilibrated with equilibration buffer (50 mM Tris-Cl pH 8.0 and 150 mM NaCl and was then calibrated with the standard proteins of known molecular mass. The standard proteins used were β-amylase (200 kDa), alcohol dehydrogenase (158 kDa), albumin (66 kDa), carbonic anhydrase (29 kDa), and cytochrome C (12.4 kDa) (Sigma, catalog no. MWGF-200). Around 250 μg rLinCas2C and rLinCas2C_Lai were resolved in SEC.

***Immunoblotting.*** Recombinant LinCas2C, LinCas2B, and LinCas2C_Lai (50 ng each) were immunoblotted as described before [4]. Polyclonal anti-LinCas2C and anti-LinCas2B were used at 1:1000 dilution, and anti-mouse HRPO conjugated secondary antibody was used at 1:5000 dilution. Analysis of native LinCas2C in *L. interrogans* serovar Copenhageni lysate was performed as described before [5].

***Nuclease assay.*** Nuclease activity of rLinCas2C_Lai was investigated on various DNA and RNA substrates. The DNA substrate used was circular double-stranded (ds) plasmid DNA (pET-28a, 0.5µg), circular single-stranded (ss) DNA (M13mp18, 0.5µg) and linear ssDNA (Φx174 genome, 0.5µg). The firefly *luciferase* mRNA (0.5µg) was used as an RNA substrate. These substrates were independently incubated with rLinCas2C_Lai in a 25 μl reaction buffer (25 mM Tris-HCl pH 8.0, 100 mM KCl, and 2.5 mM MgCl_2_) at 37°C for an hour. Divalent metal ion (2.5 mM) dependence for DNase activity was determined with various divalent metal ions (MgCl_2_, MnSO_4_, CaCl_2_, NiSO_4_, FeSO_4_, CuSO_4_ and ZnSO_4_). The reaction products were electrophoresed on ethidium bromide-stained 2% (w/v) agarose gel.

**Legends to Supplementary figures**

**Figure S1: Purification and immunoassay of rLinCas2C or its variants and rLinCas2C_Lai.** (A) Purified recombinant LinCas2C or its variant and LinCas2C_Lai resolved on a polyacrylamide gel. Each protein was overexpressed in *E. coli* BL21 (DE3 cells) using 1 mM IPTG at 37˚C for 4 hours and purified using Ni-NTA affinity chromatography in native condition. Proteins were resolved on 15% SDS-PAGE and stained with Coomassie Blue. (B) Size-exclusion chromatography of rLinCas2C and rLinCas2C_Lai. Chromatogram shows the rLinCas2C eluted at the dimeric (approx. 28 kDa) and monomeric size (approx.15 kDa), whereas LinCas2C_Lai eluted at higher oligomeric size (approx. 34 kDa) along with monomeric size (approx. 12 kDa). Standard proteins like β-amylase (200 kDa), alcohol dehydrogenase (158 kDa), albumin (66 kDa), carbonic anhydrase (29 kDa) and cytochrome C (12.4 kDa) were used to determine the protein size. (C) Immunoblot to detect rLinCas2C and rLinCas2C_Lai with anti-LinCas2B. Primary antibody was diluted at 1:1000 and the HRP-conjugated anti-rabbit secondary antibody at 1:5000. (D) Immunoblot to detect rLinCas2C and rLinCas2C_Lai with anti-LinCas2C. Primary antibodies were diluted at 1:1000 and HRP-conjugated anti-mouse secondary antibodies at 1:5000. (E) Detection of native LinCas2C expression in *L. interrogans* serovar Copenhageni (L.i sv) by immunoblot. *Leptospira* lysates were probed with anti-rLinCas2C (1:1000 dilution) and HRP-conjugated anti-mouse secondary antibody (1:5000 dilution).

**Figure S2: Nuclease activity of rLinCas2C_Lai on DNA and RNA.** DNase and RNase activity was carried out at 37ºC for an hour. (A) Concentration-dependent DNase activity of recombinant LinCas2C_Lai on plasmid-1 of 5.3 kb (pET28a, 0.5µg) in the presence of Mg^2+^ ion. Complete cleavage of the substrate was observed at 25 µM of LinCas2C. (B) DNase activity of rLinCas2C_Lai in the presence of different divalent metal ions on plasmid exemplifies its optimum activity in Mg^2+^ and Mn^2+^ ions. (C) DNase activity of rLinCas2C_Lai on linear single-stranded DNA (0.5µg of 6.4 kb M13mp18). Complete degradation of linear single-stranded was observed in the presence of Mg^2+^ ions. (D) DNase activity of rLinCas2C_Lai on circular single-stranded DNA (3.6 kb ϕx175, 0.5µg). Complete degradation of circular single-stranded was observed in the presence of Mg^2+^ ion. (E) RNase activity of rLinCas2C_Lai on *luciferase* mRNA (0.5µg). DNA ladder: 2 log DNA ladder (NEB). rLinCas2C_Lai: 25 µM and Mg^2+^: 2.5 mM. The nuclease reaction products were analyzed on 2% agarose gel.

**Figure S3: Modelled structure of LinCas2C_Lai and superimposition with its orthologs.** (A) Modeled structure of LinCas2C_Lai obtained by the homology modeling via I-TASSER program using the template of SpyCas2 from *Streptococcus pyogenes* serotype M1 (4QR0) with rmsd of 0.6 Å. (B) Structure correlation of LinCas2C_Lai with SpyCas2, (C) BhaCas2; rmsd: 0.7 Å, (D) DvuCas2; rmsd: 1.7 Å, (E) SsoCas2; rmsd: 0.8 Å.

**Figure S4: Recombinant LinCas2C and nucleic acids interaction analysis**. The interaction of rLinCas2C with non-specific DNA was analyzed by the NPDock webserver. Amino acid residues of rLinCas2C interacting with DNA at a distance of ≤ 3.5 Å are labeled.

**Table S1.** Oligos used in this work.

| **Sequence (5' -3')** | **Purpose** |
| --- | --- |
| F: CGCGGATCCCATGTTTATCATTGTATGTTACGACGT | LinCas2C forward (*BamH*I) and reverse (*Sal*I) primer |
| R: GCGTCGACTTAAAAATCAAGAATGTTAGAAACTCC |  |
| F: CATTGTATGTgcaGACGTAGAGACGATTAC | LinCas2C^Y7A^ forward and reverse primer |
| R: ATAAACATTTAAAAATCAAGAATGTTAG |  |
| F: CATTGTATGTgcagccGTAGAGACGATTACCC | LinCas2C^Y7A+D8A^ forward and reverse primer |
| R: ATAAACATTTAAAAATCAAGAATGTTAG |  |
| F: atccgttgcaGAATGCCAACTGGAACCAG | LinCas2C^R33A+F39A^ forward and reverse primer |
| R: ttttgaactgcTTGGCCATGGCTTTCGCA |  |
| F: AATCTTAGAATCTATTCTCTCG | LinCas2C^ΔL2^ forward and reverse primer |
| R: TATAATTTTAGAAAGTTTTGCTTC |  |
| CCGAACTTTCAATTCTATAAGAG | Substrate S3 (23-mer oligo) |
| TTTTTTTTTTTTTTATTATCTGAGGGTTTAATCTTATTAATCTCTTACTA | Substrate S4 (50-mer oligo) |

**Table S2.** Residues of rLinCas2C protomer A interacting with protomer B (≤ 3.5 Å).

| **Protomer A** | **Residues** | **Protomer B** | **Residues** | **No. of H-bond** |
| --- | --- | --- | --- | --- |
| β-1 | Asp8 | Turn | Lys36 | 1 |
| β-2 | Gln35 | β-1, β-4 | Cys6 and Asn65 | 1,2 |
| Turn | Lys36 | β-1 | Asp8 | 1 |
| α-2 | Glu52 | β-5 | Lys80 | 1 |
| Loop 2 | Thr63 | Turn | Asn86 | 1 |
| Loop 2 | Asp64 | β-5 | Val84 | 1 |
| β-4 | Asn65 | β-2, β-4 | Gln35, Val84 | 2,1 |
|  | Leu66 | β-5 | Phe82 | 2 |
|  | Arg67 | β-3, β-5 | Glu40, Gln81 | 2,1 |
|  | Ile68 | β-5 | Lys80 | 2 |
|  | Ser70 |  | Lys78, Lys80 | 1,1 |
| α-3 | Ser75 | β-4 | Asp72 | 2 |
| β-5 | Ser77 | β-4 | Ser70 | 2 |
|  | Lys78 |  | Ser70 | 1 |
|  | Lys80 |  | Ile68 | 2 |
|  | Val84 | Loop2, β-4 | Asp64 and Asn65 | 1,1 |
|  | Phe82 | β-4 | Leu66 | 2 |
|  | Gln81 |  | Asn65, Arg67 | 1,1 |

**Table S3.** Residues of rLinCas2C protomers interacting with ds-DNA (distance of ≤ 3.5$Å$).

| **Region** | **Protomer A** | **Protomer B** |
| --- | --- | --- |
| β1 | Asp8 | Asp8 |
| Loop 1 | Val9 | Val9 |
|  | Glu10 | Glu10 |
|  | Thr11 | Thr11 |
|  | Ile12 | Ile12 |
|  | Thr13 | - |
| α1 | Gln14 | Gln14 |
|  | Arg17 | Arg17 |
|  | Leu20 | Leu20 |
|  | Arg21 | - |
| β2 | Arg33 | Arg33 |
| Turn | Lys36 | Lys36 |
| Loop 2 | Asn60 | Asn60 |
|  | Lys62 | Lys62 |
|  | Thr63 | Thr63 |
|  | Asp64 | Asp64 |
|  | Asn86 | - |

***References.***

[1] B. Dixit, K.K. Ghosh, G. Fernandes, P. Kumar, P. Gogoi, M. Kumar, Dual nuclease activity of a Cas2 protein in CRISPR–Cas subtype I‐B of Leptospira interrogans, Febs Letters 590(7) (2016) 1002-1016.

[2] B. Dixit, V. Anand, M.S. Hussain, M. Kumar, The CRISPR-associated Cas4 protein from Leptospira interrogans demonstrate versatile nuclease activity, Current Research in Microbial Sciences (2021) 100040.

[3] A. Prakash, M. Kumar, Characterizing the transcripts of Leptospira CRISPR IB array and its processing with endoribonuclease LinCas6, International Journal of Biological Macromolecules 182 (2021) 785-795.

[4] A. Dhara, M.S. Hussain, D. Datta, M. Kumar, Insights to the assembly of a functionally active leptospiral ClpP1P2 protease complex along with its ATPase chaperone ClpX, ACS omega 4(7) (2019) 12880-12895.

[5] B. Dixit, A. Prakash, P. Kumar, P. Gogoi, M. Kumar, The core Cas1 protein of CRISPR-Cas IB in Leptospira shows metal-tunable nuclease activity, Current research in microbial sciences 2 (2021) 100059.
