# Supplementary material for: Structural and functional characterization of Cas2 of CRISPR-Cas subtype I-C lacking the CRISPR component
Source: Front Mol Biosci. 2022 Sep 12;9:988569. doi: 10.3389/fmolb.2022.988569 (PMC9510766; doi:10.3389/fmolb.2022.988569)
Supplement: Supplementary file 2 [file DataSheet1.pdf]

Supplementary information

Structural and functional characterization of Cas2 of CRISPR-Cas subtype I-C lacking the CRISPR component

Vineet Anand*, Harshini Sheeja Prabhakaran*, Prerana Gogoi, Shankar Prasad Kanaujia#, and Manish Kumar#

Department of Biosciences and Bioengineering, Indian Institute of Technology Guwahati, Guwahati -781039, Assam, India

*Equal contribution

#corresponding authors:

Manish Kumar and Shankar Prasad Kanaujia

Department of Biosciences and Bioengineering, Indian Institute of Technology Guwahati, Guwahati-781039, Assam, India

Email: mkumar1@iitg.ac.in

Phone: +91-361-258-2230

Fax: +91-361-258-2249

Running Title: Structure of LinCas2C and its nuclease activity

Materials and Methods

Protein overexpression and purification. The recombinant LinCas2C were overexpressed in *E. coli* BL21 (DE3) cells with 1 mM isopropyl β-D-1-thiogalactopyranoside (IPTG) at 37ºC for 4 h. The purification of rLinCas2C and rLinCas2C_Lai was initially carried out in native conditions. However, rLinCas2C_Lai was expressed in truncated form (8.6 kDa) and remained insoluble in native conditions. Therefore, rLinCas2C_Lai was purified using the hybrid method described before [1-3]. Purified rLinCas2C and LinCas2C_Lai was dialysed against protein storage buffer (25 mM Tris-Cl (pH 8.0), 100 mM NaCl and 10% glycerol). The yield of rLinCas2C and rLinCas2C_Lai purification was 5 mg and 40 μg per liter, respectively, and the purified proteins were stored at -20° till further use. Polyclonal antibody was generated against rLinCas2C as described before [4].

Size-exclusion chromatography. Size-exclusion chromatography was performed using Superdex 200 increase column (GE Healthcare, catalog. no.28-9909-44) on AKTA prime plus (GE Healthcare). The column was equilibrated with equilibration buffer (50 mM Tris-Cl pH 8.0 and 150 mM NaCl and was then calibrated with the standard proteins of known molecular mass. The standard proteins used were β-amylase (200 kDa), alcohol dehydrogenase (158 kDa), albumin (66 kDa), carbonic anhydrase (29 kDa), and cytochrome C (12.4 kDa) (Sigma, catalog no. MWGF-200). Around 250 μg rLinCas2C and rLinCas2C_Lai were resolved in SEC.

Immunoblotting. Recombinant LinCas2C, LinCas2B, and LinCas2C_Lai (50 ng each) were immunoblotted as described before [4]. Polyclonal anti-LinCas2C and anti-LinCas2B were used at 1:1000 dilution, and anti-mouse HRPO conjugated secondary antibody was used at 1:5000 dilution. Analysis of native LinCas2C in *L. interrogans* serovar Copenhageni lysate was performed as described before [5].

Nuclease assay. Nuclease activity of rLinCas2C_Lai was investigated on various DNA and RNA substrates. The DNA substrate used was circular double-stranded (ds) plasmid DNA (pET-28a, 0.5μg), circular single-stranded (ss) DNA (M13mp18, 0.5μg) and linear ssDNA (Φx174 genome, 0.5μg). The firefly *luciferase* mRNA (0.5μg) was used as an RNA substrate. These substrates were independently incubated with rLinCas2C_Lai in a 25 μl reaction buffer (25 mM Tris-HCl pH 8.0, 100 mM KCl, and 2.5 mM $MgCl_2$) at 37°C for an hour. Divalent metal ion (2.5 mM) dependence for DNase activity was determined with various divalent metal ions ($MgCl_2$, $MnSO_4$, $CaCl_2$, $NiSO_4$, $FeSO_4$, $CuSO_4$ and $ZnSO_4$). The reaction products were electrophoresed on ethidium bromide-stained 2% (w/v) agarose gel.

Legends to Supplementary figures

Figure S1: Purification and immunoassay of rLinCas2C or its variants and rLinCas2C_Lai. (A) Purified recombinant LinCas2C or its variant and LinCas2C_Lai resolved on a polyacrylamide gel. Each protein was overexpressed in *E. coli* BL21 (DE3 cells) using 1 mM IPTG at 37˚C for 4 hours and purified using Ni-NTA affinity chromatography in native condition. Proteins were resolved on 15% SDS-PAGE and stained with Coomassie Blue. (B) Size-exclusion chromatography of rLinCas2C and rLinCas2C_Lai. Chromatogram shows the rLinCas2C eluted at the dimeric (approx. 28 kDa) and monomeric size (approx.15 kDa), whereas LinCas2C_Lai eluted at higher oligomeric size (approx. 34 kDa) along with monomeric size (approx. 12 kDa). Standard proteins like β-amylase (200 kDa), alcohol dehydrogenase (158 kDa), albumin (66 kDa), carbonic anhydrase (29 kDa) and cytochrome C (12.4 kDa) were used to determine the protein size. (C) Immunoblot to detect rLinCas2C and rLinCas2C_Lai with anti-LinCas2B. Primary antibodies were diluted at 1:1000 and the HRP-conjugated anti-rabbit secondary antibodies at 1:5000. (D) Immunoblot to detect rLinCas2C and rLinCas2C_Lai with anti-LinCas2C. Primary antibodies were diluted at 1:1000 and HRP-

conjugated anti-mouse secondary antibodies at 1:5000. (E) Detection of native LinCas2C expression in *L. interrogans* serovar Copenhageni (L.i sv) by immunoblot. *Leptospira* lysates were probed with anti-rLinCas2C (1:1000 dilution) and HRP-conjugated anti-mouse secondary antibodies (1:5000 dilution).

Figure S2: Nuclease activity of rLinCas2C_Lai on DNA and RNA. DNase and RNase activity was carried out at 37ºC for an hour. (A) Concentration-dependent DNase activity of recombinant LinCas2C_Lai on plasmid-1 of 5.3 kb (pET28a, 0.5µg) in the presence of Mg^{2+} ion. Complete cleavage of the substrate was observed at 25 µM of LinCas2C. (B) DNase activity of rLinCas2C_Lai in the presence of different divalent metal ions on plasmid exemplifies its optimum activity in Mg^{2+} and Mn^{2+} ions. (C) DNase activity of rLinCas2C_Lai on linear single-stranded DNA (0.5µg of 6.4 kb M13mp18). Complete degradation of linear single-stranded was observed in the presence of Mg^{2+} ions. (D) DNase activity of rLinCas2C_Lai on circular single-stranded DNA (3.6 kb Φx174, 0.5µg). Complete degradation of circular single-stranded was observed in the presence of Mg^{2+} ion. (E) RNase activity of rLinCas2C_Lai on *luciferase* mRNA (0.5µg). DNA ladder: 2 log DNA ladder (NEB). rLinCas2C_Lai: 25 µM and Mg^{2+}: 2.5 mM. The nuclease reaction products were analyzed on 2% agarose gel.

Figure S3: Modelled structure of LinCas2C_Lai and superimposition with its orthologs. (A) Modeled structure of LinCas2C_Lai obtained by the homology modeling via I-TASSER program using the template of SpyCas2 from *Streptococcus pyogenes* serotype M1 (4QR0) with rmsd of 0.6 Å. (B) Structure correlation of LinCas2C_Lai with SpyCas2, (C) BhaCas2; rmsd: 0.7 Å, (D) DvuCas2; rmsd: 1.7 Å, (E) SsoCas2; rmsd: 0.8 Å.

Figure S4: Recombinant LinCas2C and nucleic acids interaction analysis. The NPDock webserver analyzed the interaction of rLinCas2C with non-specific DNA. Amino acid residues of rLinCas2C interacting with DNA at a distance of ≤ 3.5 Å are labeled.

Table S1. Oligos used in this work

Sequence (5' -3')	Purpose
F: CGCGGATCCCATGTTTATCATTGTATGTTACGACGT	LinCas2C forward (*BamH*I) and reverse (*Sal*I) primer
R: GCGTCGACTTAAAAATCAAGAATGTTAGAAACTCC	
F: CATTGTATGTgcaGACGTAGAGACGATTAC	LinCas2C^{Y7A} forward and reverse primer
R: ATAAACATTTAAAAATCAAGAATGTTAG	
F: CATTGTATGTgcagccGTAGAGACGATTACCC	LinCas2C$^{Y7A+D8A}$ forward and reverse primer
R: ATAAACATTTAAAAATCAAGAATGTTAG	
F: atccgttgcaGAATGCCAACTGGAACCAG	LinCas2C$^{R33A+F39A}$ forward and reverse primer
R: ttttgaactgcTTGGCCATGGCTTTCGCA	
F: AATCTTAGAATCTATTCTCTCG	LinCas2C$^{\Delta L2}$ forward and reverse primer
R: TATAATTTTAGAAAGTTTTGCTTC	
CCGAACTTTCAATTCTATAAGAG	Substrate S3 (23-mer oligo)
TTTTTTTTTTTTTTATTATCTGAGGGTTTAATCTTATTAATCT CTTACTA	Substrate S4 (50-mer oligo)

Table S2. Residues of rLinCas2C protomer A interacting with protomer B (≤ 3.5 Å).

Protomer A	Residues	Protomer B	Residues	No. of H-bond
β-1	Asp8	Turn	Lys36	1
β-2	Gln35	β-1, β-4	Cys6 and Asn65	1,2
Turn	Lys36	β-1	Asp8	1
α-2	Glu52	β-5	Lys80	1
Loop 2	Thr63	Turn	Asn86	1
Loop 2	Asp64	β-5	Val84	1
β-4	Asn65	β-2, β-4	Gln35, Val84	2,1
	Leu66	β-5	Phe82	2
	Arg67	β-3, β-5	Glu40, Gln81	2,1
	Ile68	β-5	Lys80	2
	Ser70		Lys78, Lys80	1,1
α-3	Ser75	β-4	Asp72	2
β-5	Ser77	β-4	Ser70	2
	Lys78		Ser70	1
	Lys80		Ile68	2
	Val84	Loop2, β-4	Asp64 and Asn65	1,1
	Phe82	β-4	Leu66	2
	Gln81		Asn65, Arg67	1,1

Table S3. Residues of rLinCas2C protomers interacting with ds-DNA (distance of ≤ 3.5 Å).

**Region**	**Protomer A**	**Protomer B**
β1	Asp8	Asp8
Loop 1	Val9	Val9
	Glu10	Glu10
	Thr11	Thr11
	Ile12	Ile12
	Thr13	-
α1	Gln14	Gln14
	Arg17	Arg17
	Leu20	Leu20
	Arg21	-
β2	Arg33	Arg33
Turn	Lys36	Lys36
Loop 2	Asn60	Asn60
	Lys62	Lys62
	Thr63	Thr63
	Asp64	Asp64
	Asn86	-

References.

[1] B. Dixit, K.K. Ghosh, G. Fernandes, P. Kumar, P. Gogoi, M. Kumar, Dual nuclease activity of a Cas2 protein in CRISPR–Cas subtype I□B of Leptospira interrogans, Febs Letters 590(7) (2016) 1002-1016.

[2] B. Dixit, V. Anand, M.S. Hussain, M. Kumar, The CRISPR-associated Cas4 protein from Leptospira interrogans demonstrate versatile nuclease activity, Current Research in Microbial Sciences (2021) 100040.

[3] A. Prakash, M. Kumar, Characterizing the transcripts of Leptospira CRISPR IB array and its processing with endoribonuclease LinCas6, International Journal of Biological Macromolecules 182 (2021) 785-795.

[4] A. Dhara, M.S. Hussain, D. Datta, M. Kumar, Insights to the assembly of a functionally active leptospiral ClpP1P2 protease complex along with its ATPase chaperone ClpX, ACS omega 4(7) (2019) 12880-12895.

[5] B. Dixit, A. Prakash, P. Kumar, P. Gogoi, M. Kumar, The core Cas1 protein of CRISPR-Cas IB in Leptospira shows metal-tunable nuclease activity, Current research in microbial sciences 2 (2021) 100059.
